# Loss-of-function maternal-effect mutations of *PADI6* are associated with familial and sporadic Beckwith-Wiedemann syndrome with multi-locus imprinting disturbance

**DOI:** 10.1186/s13148-020-00925-2

**Published:** 2020-09-14

**Authors:** Maria Vittoria Cubellis, Laura Pignata, Ankit Verma, Angela Sparago, Rosita Del Prete, Maria Monticelli, Luciano Calzari, Vincenzo Antona, Daniela Melis, Romano Tenconi, Silvia Russo, Flavia Cerrato, Andrea Riccio

**Affiliations:** 1 Department of Biology, Università degli Studi di Napoli “Federico II”, Napoli, Italy; 2 Department of Environmental Biological and Pharmaceutical Sciences and Technologies (DiSTABiF), Università degli Studi della Campania “Luigi Vanvitelli”, Caserta, Italy; 3 Institute of Genetics and Biophysics (IGB) “Adriano Buzzati-Traverso”, Consiglio Nazionale delle Ricerche (CNR), Naples, Italy; 4 Medical Cytogenetics and Molecular Genetics Laboratory, Centro di Ricerche e Tecnologie Biomediche IRCCS, Istituto Auxologico Italiano, Milan, Italy; 5 Department of Sciences for Health Promotion and Mother and Child Care “G. D’Alessandro”, University of Palermo, Palermo, Italy; 6 Medical, Surgical, and Dental Department, Università degli Studi di Salerno, Salerno, Italy; 7 Department of Pediatrics, Clinical Genetics, Università di Padova, Padova, Italy

**Keywords:** Multi-locus imprinting disturbance, *PADI6*, Beckwith-Wiedemann syndrome, Genomic imprinting, DNA methylation, Maternal-effect variants, Subcortical maternal complex, Infertility

## Abstract

**Background:**

PADI6 is a component of the subcortical maternal complex, a group of proteins that is abundantly expressed in the oocyte cytoplasm, but is required for the correct development of early embryo. Maternal-effect variants of the subcortical maternal complex proteins are associated with heterogeneous diseases, including female infertility, hydatidiform mole, and imprinting disorders with multi-locus imprinting disturbance. While the involvement of *PADI6* in infertility is well demonstrated, its role in imprinting disorders is less well established.

**Results:**

We have identified by whole-exome sequencing analysis four cases of Beckwith-Wiedemann syndrome with multi-locus imprinting disturbance whose mothers are carriers of *PADI6* variants. In silico analysis indicates that these variants result in loss of function, and segregation analysis suggests they act as either recessive or dominant-negative maternal-effect mutations. Genome-wide methylation analysis revealed heterogeneous and extensively altered methylation profiles of imprinted loci in the patients, including two affected sisters, but not in their healthy siblings.

**Conclusion:**

Our results firmly establish the role of *PADI6* in imprinting disorders. We report loss-of-function maternal-effect variants of *PADI6* that are associated with heterogeneous multi-locus imprinting disturbances in the progeny. The rare finding of two siblings affected by Beckwith-Wiedemann syndrome suggests that in some cases, familial recurrence risk of these variants may be high. However, the heterogeneous phenotypes of the other pedigrees suggest that altered oocyte *PADI6* function results in stochastic maintenance of methylation imprinting with unpredictable consequences on early embryo health.

## Background

Differential DNA methylation between the maternally and paternally inherited chromosomes controls the monoallelic and parent-of-origin dependent expression of the imprinted genes, a group of about 100 loci with important roles in fetal growth, metabolism, and behavior [1]. The organization in clusters or domains of the imprinted genes enables their fine-tuned and coordinated regulation by *cis*-acting control regions. In particular, each imprinting cluster typically harbors one germline DMR (gDMR), in which differential allelic methylation is acquired during gametogenesis and is maintained throughout development in somatic cells, escaping the methylation reprogramming occurring in early embryogenesis. Currently, 38 gDMRs have been identified in the human genome and the majority of them are methylated on the maternal chromosome. Differential methylation of the gDMR is required for maintaining the parent-of-origin dependent expression of the entire imprinted gene cluster.

Genetic and epigenetic defects altering the expression of imprinted genes are associated with 12 rare clinical conditions, known as imprinting disorders [2]. Among this group of pathologies, the Beckwith-Wiedemann syndrome (BWS, OMIM #130650, prevalence of 1:10,340 live births [3]) is characterized by macrosomia, macroglossia, abdominal wall defects, neonatal hypoglycemia, lateralized overgrowth, and predisposition to Wilms tumor and other embryonal cancers [4]. At the molecular level, this disorder is caused by molecular defects affecting one or both of two imprinting domains located at chromosome 11p15.5: the telomeric domain that includes the *H19* and *IGF2* genes and is controlled by the *H19/IGF2*:IG-DMR, and the centromeric domain that includes the *KCNQ1OT1* and *CDKN1C* genes and is controlled by the *KCNQ1OT1*:TSS-DMR. The molecular defects of BWS are as follows: loss of methylation (LOM) of the *KCNQ1OT1*:TSS-DMR that is present in 50% of cases, mosaic segmental paternal unidisomy of chromosome 11p15 (upd(11)pat) in 20%, gain of methylation (GOM) of the *H19/IGF2*:IG-DMR in 5–10%, and maternal loss-of-function mutations of *CDKN1C* gene in 5% [4].

A subset of patients with imprinting disorders display methylation changes in addition to that of the locus that is normally associated with the clinical phenotype. This condition is known as multi-locus imprinting disturbance (MLID) and appears particularly frequent (one third of the cases according to ref. [4]) in BWS with *KCNQ1OT1*:TSS-DMR LOM [5, 6]. Its etiology is unknown in the majority of the cases [7]. However, in a number of families, causative loss-of-function mutations affecting either zygotic or oocyte-derived *trans*-acting factors have been found in the patients with MLID or in their mothers, respectively [1]. In particular, zygotic mutations affecting the zinc-finger *ZFP57* gene have been identified in the transient neonatal diabetes type 1 (TNDM1) [8], while maternal-effect mutations have been reported in healthy women with offspring with MLID and variable imprinting disorders including BWS, or women with reproductive problems, such as infertility, recurrent pregnancy loss, or hydatidiform mole [7]. Maternal-effect genes are abundantly expressed in oocytes but exert their phenotypic effect in the offspring, being critical for correct development of early embryos. Most of the maternal-effect genes associated with MLID or hydatidiform mole encode for components of the subcortical maternal complex (SCMC) that is localized in the periphery of the oocyte cytoplasm and early embryo until the blastocyst stage [9]. Among these genes, more compelling pieces of evidence have been provided for association of *NLRP2*, *NLRP5*, *NLRP7*, and *KHDC3L*, while the role of *PADI6*, *OOEP*, and *TLE6* in the etiology of imprinting disorders is not definitely established [7]. Although several important functions have been attributed to the SCMC during oocyte-to-embryo transition and early development (for a review, see [9]), the mechanisms through which this protein complex influences methylation imprinting remain elusive.

*PADI6* encodes a member of the peptidylarginine deiminase (PAD) family, a class of enzymes converting arginine residues to citrulline. All mammalian PADs, including PADI6, share 70–95% identity in their amino acid sequence including two immunoglobulin-like domains at the N terminus and a highly conserved C-terminal domain that harbors the active site [10]. PADI6 is the only PAD for which no enzymatic activity has been detected in vitro [11]. Mouse and human studies demonstrate that *PADI6* is highly expressed in oocytes and early embryos, where it colocalizes with the other components of the SCMC [12, 13]. *PADI6* is required for the formation of the oocyte lattices that are believed to work as ribosomal storage for early embryo [14]. Indeed, the development of *Padi6*-null embryos is arrested at the 2-cell stage, and their ribosomal components, de novo protein synthesis, and embryonic genome activation are impaired [15]. In humans, homozygous loss-of-function mutations of *PADI6* are associated with female infertility and hydatidiform mole (Additional file 1: Table S1) [13, 16–19]. In addition, biallelic or monoallelic missense *PADI6* variants have been linked with three cases of Silver-Russell syndrome and one case of BWS [20].

Other important aspects needing clarification concern the phenotypes associated with MLID and their risk of recurrence in the families in which maternal-effect variants segregate. A few familial cases with MLID have been described so far, and highly variable expressivity and incomplete penetrance of the phenotype have been reported [20–22]. In some cases, siblings with different imprinting disorders or complex phenotypes, or pedigrees with multiple pregnancy losses and imprinting disorders were observed [20, 23].

Here, we describe four BWS cases with MLID that are associated with maternal-effect loss-of-function variants of *PADI6*. Two of the probands are siblings, indicating that maternal variants of *PADI6* may act as *trans*-acting mutations in either familial or sporadic BWS.

## Results

### Clinical cases

**Fig. 1:**
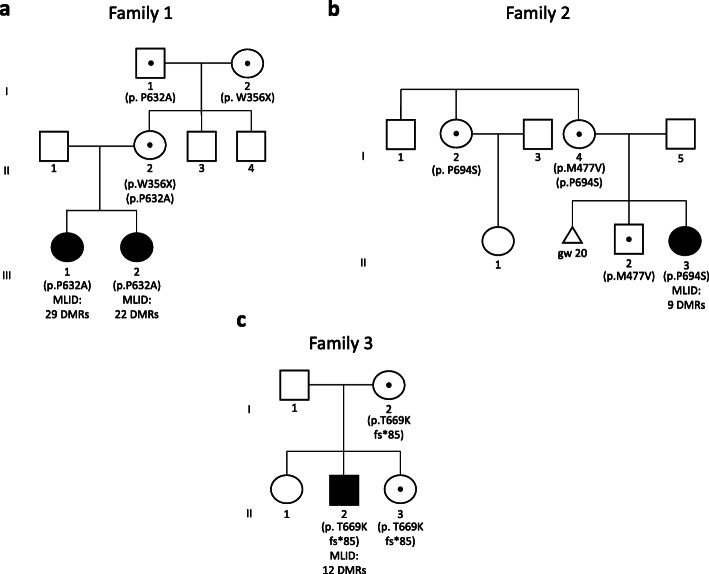
Genetic characterization of the three families under study. Pedigree and corresponding *PADI6* variants of families 1 (**a**), 2 (**b**), and 3 (**c**). Black filled symbol represents individual with BWS features, and black central dot unaffected carriers of *PADI6* variants. Weeks of gestation (gw) are reported for the aborted fetus in family 2. MLID status and the number of DMRs with altered methylation are also reported

The four patients described in this study were referred to our laboratory with clinical diagnosis of BWS [4]. Family 1 included two sisters with typical BWS features. The older one (III-1, Fig. 1a) was a 4-year-old girl born from non-consanguineous parents. She was born preterm (32 weeks of gestation) because of maternal preeclampsia and polyhydramnios, with birth weight of 1980 g (50–70th centile), length of 44 cm (70–90th centile), and cranial circumference of 31 cm (75–90th centile). At birth, she presented breathing and feeding difficulties, placental hyperplasia, and macroglossia. Hypoglycemia and anemia occurred in the perinatal period. After birth, patent foramen ovale was observed, with closure at 18 months. She also showed visual defects. Clinical examination at three and a half years revealed the presence of lateralized overgrowth of the body with the right side bigger than the left side (1 cm dysmetry), angiomas, naevus flammeus, umbilical hernia, and diastasis recti. According to the recent BWS consensus, these features correspond to a score of 8 points that is consistent with the clinical diagnosis of classical BWS [4]. Her sister (III-2, Fig. 1a) was a 1-year-old child, born at 39 weeks of gestation with placental hyperplasia and polyhydramnios. At birth, her weight was 3000 g (25–50th centile), length 48 cm (25th centile), and cranial circumference 32 cm (3–10th centile). Typical BWS features were observed, including neonatal hypoglycemia, macroglossia, naevus flammeus, and umbilical hernia. Clinical examination at 6 months revealed the presence of angiomas and patent foramen ovale. Overall, a clinical score of 6 was reached, confirming the clinical diagnosis of classical BWS, also in this case. No clinical signs of BWS were reported in the parents (II-1; II-2), grandparents (I-1 and I-2), and uncles (II-3 and II-4).

The proband of family 2 (II-3, Fig. 1b) was a 33-year-old lady born at 35 weeks of gestation from healthy unrelated parents. Birth weight was 2950 g (75–90th centile), birth length 51 cm (90–95th centile), and head circumference 34 cm (75–90th centile). She also presented diastasis recti, macroglossia, umbilical hernia, ear lobe creases, naevus flammeus, facial dysmorphism, organomegaly, renal dysplasia, and asymmetry of the chest, which allowed the clinical diagnosis of classical BWS (points = 8). Overgrowth started at the age of 6 months, no lateralized overgrowth and no feeding difficulties were observed, and normal developmental milestones were achieved. The patient was followed annually for the first 12 years of life with clinical evaluation and abdominal ultrasound. Last evaluation was at the age of 30 years. Intellectual development was normal, and she obtained a university degree. The proband’s mother had a healthy phenotype, but reported a spontaneous pregnancy loss at 20 weeks of gestation when she was 28 years old. At birth of the first son, she was 30 years old and her husband 32 years old. The proposita was born 5 years later. No conception difficulty was reported for the couple, and no clinical signs of BWS were observed in the other family members (Fig. 1b).

The proband of family 3 (II-2, Fig. 1c) was an 18-year-old male born from healthy non-consanguineous parents. He was born at 38 weeks of gestation, with birth weight of 5530 g (> 95th centile) and birth length of 54 cm (> 95th centile). During the postnatal period, he was affected by convulsions. Clinical examination at 1 year revealed the presence of macroglossia, prognathism, lateralized overgrowth, cryptorchidism, and patent foramen ovale, which together with macrosomia correspond to a clinical score of 5 that is sufficient for the clinical diagnosis of classical BWS. During follow-up, delay of language development and dorsal scoliosis were observed. More recently, because of recurrent occurrence of headache, the patient was subjected to magnetic resonance imaging, and *Arnold-Chiari* I malformation was diagnosed. The other family members showed no feature of BWS (Fig. 1c).

### DNA methylation analysis

Molecular testing for BWS was performed by DNA methylation analysis of the 11p15.5 region [4]. DNA methylation was measured by bisulfite treatment of peripheral blood leukocyte (PBL) DNA followed by pyrosequencing. *KCNQ1OT1*:TSS-DMR LOM and normal methylation of the *H19/IGF2* IG-DMR were found in all four cases, confirming the diagnosis of BWS (Additional file 2: Figure S1). Since MLID is frequently associated with *KCNQ1OT1*:TSS-DMR LOM, we extended the DNA methylation analysis to five further DMRs that are frequently hypomethylated in BWS patients [5]. The results demonstrated that all four patients had at least one additional hypomethylated DMR, thus confirming the molecular diagnosis of MLID (Additional file 2: Figure S1).

**Fig. 2:**
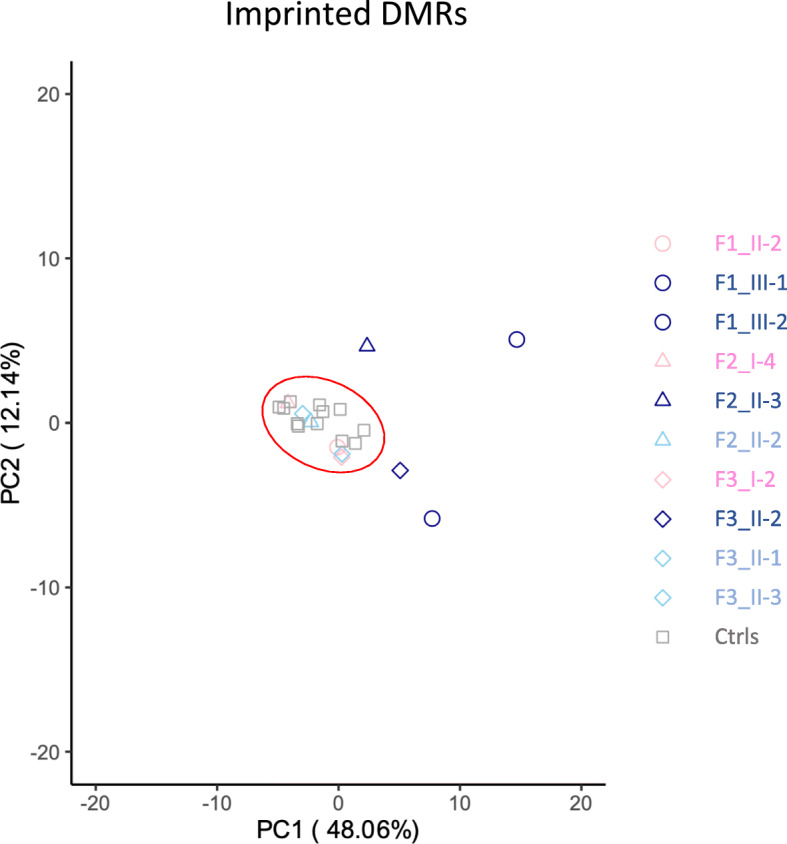
PCA of imprinted DMR methylation. DNA methylation of the imprinted DMRs of the four probands, their mothers and siblings, and 12 age-matched healthy controls was compared by PCA. Symbol code: circles represent members of family 1, triangles members of family 2, rhombi members of family 3, and squares the controls. Color code: probands are represented in dark blue, mothers in pink, siblings in light blue, controls in gray

**Fig. 3:**
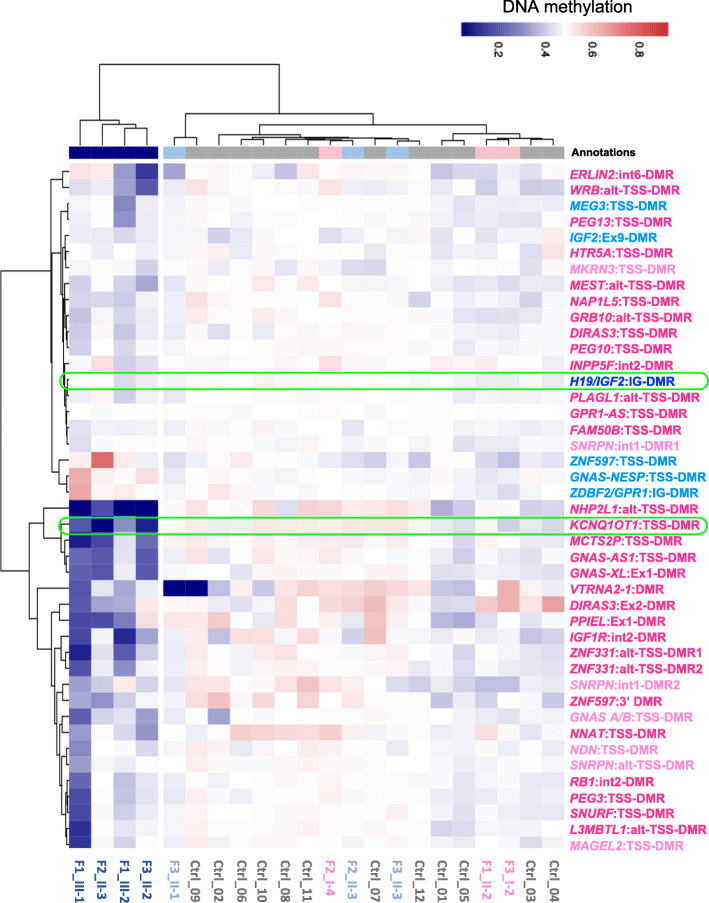
DNA methylation analysis of imprinted DMRs. Heatmap showing imprinted DMR methylation levels of the probands of families 1, 2, and 3 (F1_III-1, F1_III-2, F2_II-3, F3_II-2); their mothers (F1_II-2, F2_I-4, F3_I-2) and siblings (F2_II-2, F3_II-1, F3_II-3); and 12 age-matched control individuals, organized by hierarchical clustering. Clustering is based on CpG methylation levels of 736 probes overlapping with 43 imprinted DMRs, containing at least three CpGs. Maternally methylated germline DMRs are in dark pink; maternally methylated secondary DMRs are in light pink. Paternally methylated germline DMRs are in dark blue; paternally methylated secondary DMRs are in light blue. The *KCNQ1OT1*:TSS-DMR and the *H19/IGF2*:IG-DMR diagnostic of BWS are highlighted in green. The *VTRNA2*-1 DMR has been reported to be polymorphic [24]

To better characterize the methylation abnormalities of our patients, we determined their genome-wide methylation profiles in PBL DNAs by employing the Illumina Infinium methylation EPIC array, and compared them with those of their siblings and mothers, and 12 age-matched controls. After quality control filtering, ~ 736,000 CpG sites in each sample were retained. All these datasets were proven to be comparable after batch adjustment by principal component analysis (PCA, Additional file 3: Figure S2), and no evident difference was observed in the bimodal distribution of their genomic DNA methylation (Additional file 4: Figure S3). However, when only the CpGs underlying imprinted DMRs were considered, the four patients clustered separately from the controls and their healthy relatives, indicating that different methylation levels were present in imprinted loci (Fig. 2). To better compare the methylation profiles, we generated a heatmap (Fig. 3) depicting the methylation levels of all known imprinted DMRs [25] that were covered by at least three CpGs in our dataset. A striking difference was seen in the methylation of multiple DMRs between the four patients and controls. The methylation level of several DMRs of the patients differed in a statistically significant manner and was generally hypomethylated with respect to the controls, while only a few DMRs differed moderately in the control individuals from their average (Fig. 3 and Additional file 5: TableS2). In addition, the number and methylation level of the affected DMRs differed among the patients and even between affected siblings. Overall, the probands of family 1 had the most dramatic changes: III-1 and III-2 showed the most intense methylation changes and the highest number of affected DMRs. Overall, the most frequently affected loci in addition to *KCNQ1OT1* were *GNAS*, *MCTS2P*, *NHP2L1*, *PPIEL*, *DIRAS*, *ZNF331*, *IGF1R*, *ERLIN*, and *WRB*. Hypomethylation was observed in both maternally (e.g., *KCNQ1OT1*, *GNAS*, *MCTS2P*) and paternally methylated (e.g., *MEG3*) gDMRs. The paternally methylated secondary DMRs *GNAS*-*NESP*, *ZNF597*-TSS, and *ZDBF2/GPR1* were found hypermethylated, likely as consequence of hypomethylation of their respective maternally methylated gDMRs. A slight hypomethylation of the *KCNQ1OT1*:TSS-DMR was also evident in the mother of the family 3 proband (I-2 in Fig. 3 and Additional file 2: Fig S1c).

### Identification of maternal *PADI6* variants

**Fig. 4:**
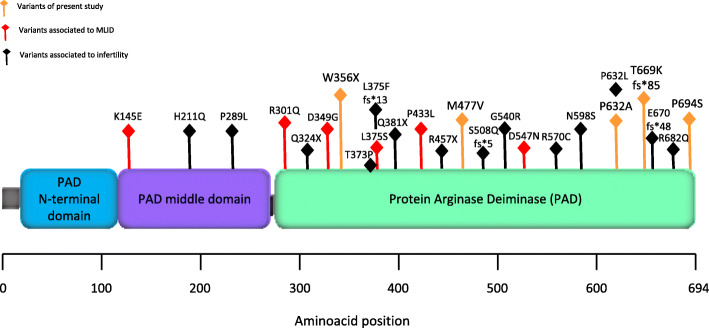
Position of the pathogenic variants in the PADI6 protein. The variants associated with infertility of the carrier women are represented with black rhombi, and variants associated with MLID in the offspring with orange or red rhombi, if described in present or previous studies, respectively

To investigate whether familial MLID was associated with genetic variants, whole-exome sequencing (WES) was performed on the DNAs of the probands and their parents of family 1. After filtering and exclusion of frequent variants, no damaging variant that was present in homozygosity or compound heterozygosity in both the probands was identified. Then, we looked for rare homozygous and compound heterozygous variants in their mother (Additional file 6: Table S3). According to the score of the mutation prediction tools (e.g., PolyPhen-2 and SIFT) of these variants and available information on the affected proteins, we identified *PADI6* as the best candidate gene causative of MLID in this family. Two single-nucleotide variants (SNVs) of *PADI6* were found in compound heterozygosity in the mother of the probands (II-2). The identified variants were a novel stop-gain mutation (chr1, g.17718714 G → A, p.W356X) in exon 10 and an extremely rare missense mutation (chr1, g.17727743 C → G, p.P632A) in exon 17, respectively (Table 1). Both variants affected the arginine deiminase domain of PADI6 (Fig. 4). P632 was conserved in orthologous proteins, and the P632A variant was predicted to be damaging by PolyPhen-2 and deleterious by SIFT (Table 1). Segregation of the variants was demonstrated by exome-seq and Sanger sequencing (Additional file 6: Table S3, Additional file 7: Figure S4a). II-2 inherited the missense variant from her father (I-1) and the stop-gain mutation from her mother (I-2). The variants were not present in the patients’ father (II-1), and only the missense variant was inherited by the probands. The presence of biallelic damaging variants in the probands’ mother indicates that they act as loss-of-function maternal-effect mutations, as previously suggested [20].

**Table 1:** List of the *PADI6* pathogenic variants and prediction of their effect on the protein structure and function

Position	Alleles	Variant ID	Allele frequency	AA	Molecular effect	Conservation in orthologous proteins	Prediction				Genotype	Family
							SIFT	Polyphen-2	SDM (ΔΔG)	Accessible surface area		
chr1 17718714	G > A	–		Trp356Ter	Nonsense	–	–	–	–		Comp het	1
chr1 17727743	C > G	rs755260464^a^	0.000004012	Pro632Ala	Missense	Yes	Deleterious	Possibly damaging	Stabilizing (0.600 kcal/mol)	*Å*^2^ = 0.61		
chr1:17721538	A > G	rs761556429^b^	0.000004008	Met477Val	Missense	Yes	Tolerated	Benign	Destabilizing (− 0.400 kcal/mol)	*Å*^2^ = 3.2	Comp het	2
chr1:17727929	C > T	rs1368496637^c^	0.000008	Pro694Ser	Missense	Yes	Deleterious	Possibly damaging	Neutral (0.000 kcal/mol)	*Å*^2^ = 61.7		
chr1:17727854-17727856	Del C	–		Thr669Lys	fs*85	–	–	–	–		Het	3

Allele frequency was worldwide, as reported by GnomeAD

*Comp het* compound heterozygous, *Het* heterozygous

^a^Observed only in heterozygosity in a Swedish male

^b^Observed only in heterozygosity in a Southern European male

^c^Observed only in two males in heterozygosity

To look for further maternal-effect variants associated with MLID, WES was performed on I-4 of family 2 and I-2 of family 3. Two extremely rare missense variants (chr1, g.17727929 C → T, p.P694S; chr1, g.17721538 A → G, p.M477V) of *PADI6* were identified in compound heterozygosity in the former woman and a novel heterozygous frameshift variant (GRCh37/hg19, chr1, g.17727855 C → -, p.T669K fs*85) also in *PADI6* in the latter (Additional file 6: Table S3 and Table 1). Both P694 and M477 are conserved in orthologous proteins, and P694S is predicted to be damaging by PolyPhen-2 and deleterious by SIFT, but is considered neutral on the protein structure by the prediction tool SDM, while M477V is predicted to be benign by PolyPhen-2 and tolerated by SIFT, but internally located (accessible surface area *Å*^2^ = 3.2) and destabilizing the protein structure by SDM (Table 1). As with the variants of family 1, also those identified in families 2 and 3 affect the arginine deiminase domain of PADI6 (Fig. 4). Segregation analysis demonstrated that M477V was inherited by the healthy proband’s brother (II-2) and P694S by the proband (II-3) and her maternal aunt (I-2), in family 2 (Additional file 7: Figure S4b). In family 3, the frameshift variant pT669K fs*85 was inherited by the proband (II-2) and his healthy youngest sister (II-3, Additional file 7: Figure S4c).

**Fig. 5:**
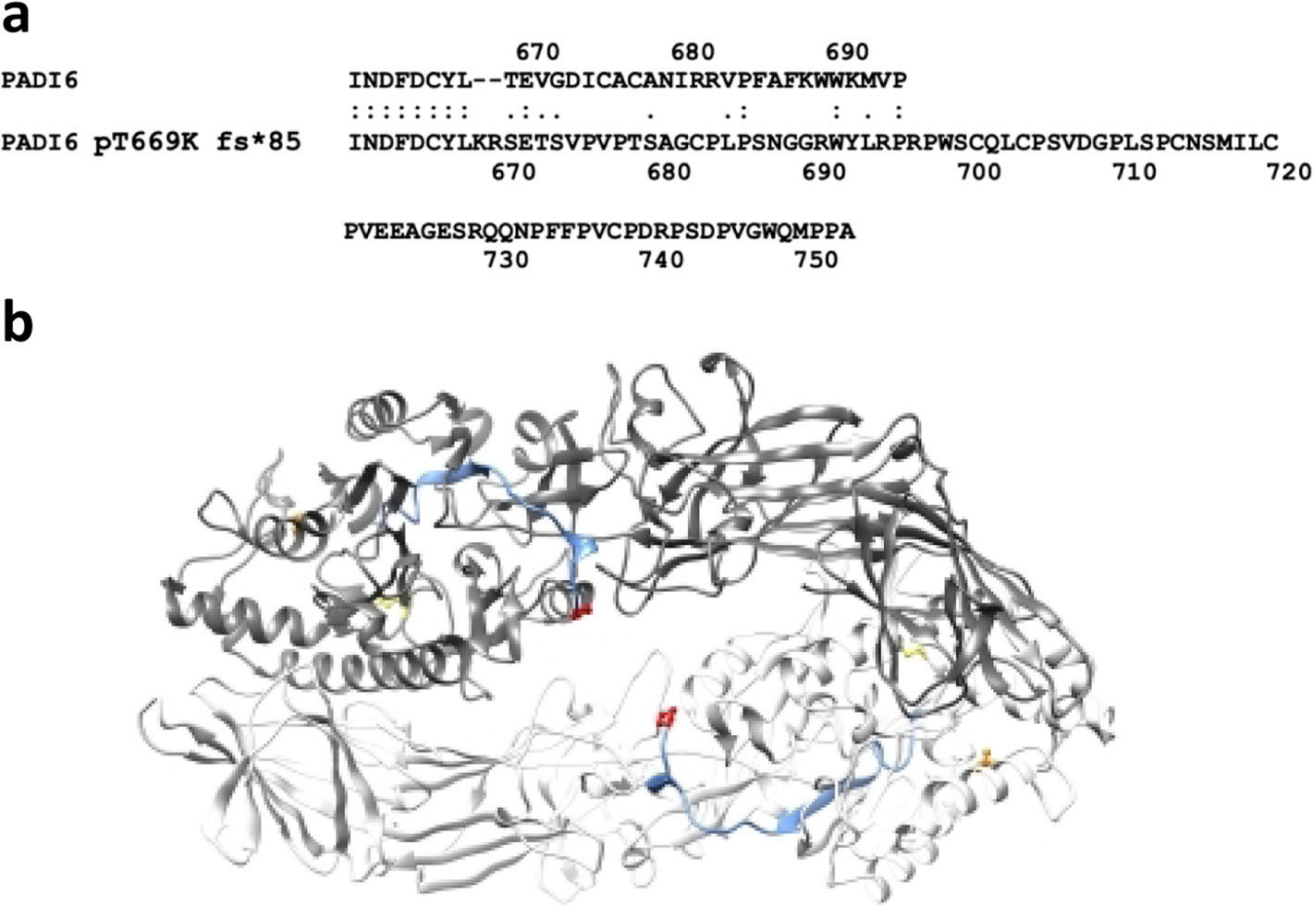
Effect of the frameshift variant on PADI6 protein structure. **a** Alignment of the C-terminal residues of the wild-type PADI6 with those of the protein resulting from the T669Kfs*85 variant. **b** Model of human wild-type PADI6 in dimeric form. The amino acids that are colored are the sites were mutations occur. The model is represented as a cartoon, and the two chains are colored in light or dark gray, respectively. The 26-aa carboxy-terminal region of the protein that is substituted by a longer peptide in the frameshift variant T669Kfs*85 is colored in blue; the side chains of M477 (yellow), P632 (orange), and P694 (red) are shown as balls-and-sticks

The frameshift T669Kfs*85 modifies the carboxy-terminal of PADI6, the last residues being substituted by a longer peptide (Fig. 5a). Since this *PADI6* variant was present in heterozygosity in I-2 of family 3, we wondered if this mutation could have a dominant effect. We generated a 3D structural model of human PADI6 by aligning it to the paralogous PADI4 that is known to be a dimer [26]. On the basis of this model, we observed that the carboxy-terminal part of PADI6 was not involved in dimerization; hence, the formation of heterodimers with dominant-negative effect in the heterozygous patient is possible (Fig. 5b).

## Discussion

DNA methylation defects of imprinting disorders arise in early embryogenesis, often as consequence of genetic mutations that act either in *cis* or in *trans*. In the latter case, MLID generally occurs in the patients [1]. Recently, a number of maternal-effect variants mostly affecting the components of the oocyte SCMC have been associated with MLID. However, the presence of heterogeneous phenotypes in the offspring of the carrier mothers makes prediction of the clinical condition and determination of recurrence risks very challenging [7]. Several studies have indicated *PADI6* variants as the cause of female infertility [16–19], but its role in imprinting disorders is less well established [20]. We report on four cases of BWS with MLID, which arise from maternal-effect loss-of-function *PADI6* mutations, and characterize their genomic methylation profiles. Our results firmly establish the role of *PADI6* in imprinting disorders and provide novel information on the recurrence risk and the extent by which these variants impact DNA methylation.

The most compelling evidence of the association of *PADI6* with BWS and MLID as maternal-effect gene is provided by our family 1. In this case, the healthy mother of two girls with BWS carries a truncating mutation and a deleterious missense variant in compound heterozygosity. The truncating variant is present also in the maternal grandmother, indicating that monoallelic inactivation of *PADI6* is not sufficient to cause the BWS phenotype in the progeny. Consistent with this observation, two damaging *PADI6* mutations have also been identified in the proband’s mother of family 2. In this case, one of the variants (M477V) was predicted as benign by PolyPhen-2 but destabilizing by a structure prediction tool, and it occurs at a site that is completely conserved in orthologous PADI6 proteins and is buried in the protein structure (Table 1). Differently from the previous cases, the frameshift *PADI6* variant affects the proband's mother of family 3 in only one allele. This variant removes the last 26 residues of the wild-type protein and substitutes them with an 85-aa-long peptide. By generating an in silico model, we predicted that if PADI6 dimerizes as the paralogous PADI4, this mutant protein would be able to heterodimerize with the wild-type isoform and possibly act as dominant-negative mutant in the heterozygous patient.

The progenies of the carrier mothers of families 2 and 3 included both affected and healthy siblings. Also, the proband’s mother of family 3 showed slight *KCNQ1OT1* hypomethylation suggesting maternal inheritance of the frameshift variant, but she had no feature of BWS. Incomplete penetrance of the clinical phenotype associated with maternal variants of *PADI6* is also evident in two previously reported pedigrees [20] and is consistent with the findings on other SCMC members [20, 23]. This could be due to partial loss of protein activity resulting in mosaic and heterogeneous alteration of DNA methylation in early embryos. Therefore, although the presence of two affected siblings in family 1, and a BWS patient and a miscarriage in family 2, suggests that in some cases recurrence risk of maternal SCMC variants may be high, this is not always the case, and analysis of larger cohorts is needed for better predictions.

Our genome-wide methylation analysis revealed that the MLID associated with maternal *PADI6* variants may involve many imprinted loci. Strikingly, in the most intensely affected patient (III-1 of family 1), up to 50% of the investigated DMRs showed abnormal methylation. Interestingly, the methylation profile differed among patients and was extremely heterogeneous even in the affected siblings of family 1. Both maternally methylated and paternally methylated imprinted DMRs were found hypomethylated in the patients, suggesting that the methylation defects arise post-fertilization. Some secondary DMRs were hypermethylated, consistent with the described hierarchical control by their respective gDMR [1]. Also, both hypo- and hypermethylation were present at variable level, indicating that the methylation disturbances were mosaic.

It is possible that the methylation abnormalities of loci other than *KCNQ1OT1* may modify the typical clinical picture of BWS [22, 27–29]. Consistent with this hypothesis, we observe some clinical features (e.g., absence of macrosomia at birth) that are infrequent in BWS. It is also worth to mention that three of the four MLID cases with *PADI6* variants described so far displayed a Silver-Russell syndrome (SRS) phenotype [20]. These patients have *H19/IGF2*:IG-DMR LOM, and some clinical features (feeding difficulties and body asymmetry) overlapping with the cases of the present study. This demonstrates that maternal *PADI6* variants may result in a so far unpredictable wide spectrum of DNA methylation profiles and clinical phenotypes in the offspring (Additional file 1: Table S1). These variants add further to the complexity of phenotypes associated with MLID and maternal variants so far (Additional file 8: Table S4). The great majority of these maternal variants affect SCMC protein members. However, investigation on larger cohorts is needed to establish how frequent these variants are and how large the spectra of the involved genes and phenotypes are.

Human *PADI6* variants have been associated with female infertility and hydatidiform mole [13, 16–19]. It is possible that the reproductive outcome is correlated with the severity of the *PADI6* variants. Indeed, infertility is more frequently associated with truncating variants completely disrupting the protein function or missense variants that according to SDM strongly destabilize protein structure (Additional file 1: Table S1). Conversely, MLID is more frequently associated with missense variants only partially reducing protein function (Additional file 1: Table S1). MLID-associated variants are generally non-destabilizing (e.g., P632A and P694S) or only moderately destabilizing (e.g., M477V), or a destabilizing variant is in compound heterozygosity with a non-destabilizing variant (Table 1 and Additional file 1: Table S1). Both P632A and P694S may have functional effect, because they are included in regions that are very conserved in all PAD proteins. In particular, P694 that is the last residue of the protein is part of the consensus sequence FxxWxMxP of the human C-terminome, which is known to have a functional role [30]. Concerning P632 variants, in addition to P632A we identified in MLID patients, the variant P632L has been associated with female infertility [18]. Since P632 is poorly exposed to solvent and buried inside the protein, substitution of proline with alanine may be less deleterious than substitution with leucine that has a longer side chain. In conclusion, severe loss-of-function variants may lead to female infertility, while hypomorphic variants may cause MLID. Intriguingly, in the present study, the relatively more severe maternal *PADI6* genotype (a truncating variant and a functionally damaging missense variant) is associated with a rare pedigree with two affected siblings, suggesting that severity of *PADI6* variants may also influence recurrence risk.

It is unknown how *PADI6* or the other SCMC members affect methylation imprinting. It has been recently demonstrated that *KHDC3L* that is associated with recurrent hydatidiform mole is necessary for de novo methylation in human oocytes [31]. *NLRP2*, *NLRP5*, and *PADI6* that are associated with MLID and mosaic hypomethylation of both maternal and paternal gDMRs more likely affect post-zygotic imprinting maintenance. Although it is unclear if PADI6 has catalytic activity, most of the pathogenic missense variants of *PADI6* identified so far affect its arginine deiminase domain (Fig. 4), indicating an important role of this domain for protein function. It is possible that imprinting disturbances arise as consequence of defective storage, de novo synthesis, cellular localization, or inappropriate degradation of several maternal-zygotic factors including those controlling DNA methylation maintenance in early embryos. Our study suggests that hypomorphic *PADI6* mutations cause MLID, while complete loss-of-function variants lead to female infertility. Thus, further functional studies reproducing the hypomorphic variants that are present in the MLID patients may help clarifying the role of SCMC in the maintenance of epigenome integrity.

## Conclusions

In summary, we investigated the etiology of MLID cases associated with BWS. Using WES analysis, we identified several loss-of-function variants of *PADI6* that segregated with MLID and BWS, as maternal-effect mutations. Further, we showed that the genomic methylation profiles of the MLID patients with *PADI6* variants can be extensively altered but also very heterogeneous. Overall, our data suggest that maternal *PADI6* is necessary for methylation maintenance in early embryos and that its deficiency results in stochastic imprinted disturbances that may variably impact the health of the progeny.

## Methods

### Patient cohort

The four patients described in this study were part of a cohort of 72 BWS patients with *KCNQ1OT1*:TSS-DMR LOM. Of these, 18 (25%) were demonstrated to have MLID after testing 7 DMRs by pyroseq, COBRA, or MS-MLPA. In four pedigrees we have sent out for exome sequencing so far, maternal *NLRP5* variants were identified in one family [22] and *PADI6* variants in the other 3 families (present study).

### Ethics

Genetic analyses were performed after written informed consent was obtained from the patients or patients’ parents. The research work was carried out in accordance with the ethical principles and the Italian legislation. The study was approved by the Ethical Committee of the University of Campania “Luigi Vanvitelli” (Naples, Italy; approval number: 1135, 13 October 2016).

### Genomic DNA extraction

Genomic DNA was isolated from peripheral blood lymphocytes (PBLs) by using the salting-out procedure ([32]; Nucleic acids research). Nucleic acid concentration was determined by using NanoDrop spectrophotometer (Termo Fisher Scientific, Italy).

### DNA methylation analysis

#### Locus-specific analysis by pyrosequencing

One and a half micrograms of PBL DNA was treated with sodium bisulfite by using the EpiTect Bisulfite kit (Qiagen-Italia, Milan, Italy) following the manufacturer’s protocol. About 200 ng of converted DNA was amplified by using the PyroMark PCR kit (Qiagen-Italia, Milan, Italy) in a final volume of 25 μl. Fifteen microliters of PCR product was used for quantitative DNA methylation by pyrosequencing on a Pyromark Q48 Autoprep system with the PyroMark Q48 Adv. CpG Reagents Kit (Qiagen-Italia, Milan, Italy) and PyroMark Q48 Magnetic Beads. Results were analyzed by using the Pyromark Q48 Autoprep software. The primers, used for PCR amplification and sequencing, were designed with Pyromark Assay Design SW 2.0 and reported in Additional file 9: Table S5.

#### Genome-wide methylation array profiling

Genomic DNA was extracted from PBL of probands, siblings, mothers, and 12 controls (whose sex, gender, and age are listed in Additional file 10: Table S6) and subjected to bisulfite conversion methylation array processing as described previously [22]. Data was analyzed using R version 3.6.1. Beta values were extracted from “idat” files by using the “champ.load” module of the “Champ” R package (v. 2.16.2), with quality control options set as default and array type as “EPIC.” The quality control step retained 736,048 probes, which were used for further analysis. Probes overlapped with SNP (96,368) and with a detection *p* value < 0.01 (3456), bead count < 3 in at least 5% of samples in one or more samples (10,453) were eliminated. X or Y chromosome probes (16,664), non-cg probes (2918), and probes aligning to multiple locations (11) and unannotated probes were also removed from analysis. No background correction was carried out. BMIQ normalization was applied, with the default options for array type as “EPIC.” BMIQ normalized samples were assigned with respective genome coordinates using B4 annotation of Illumina manifest file (hg19) (Illumina Inc., USA). Since six out of twelve controls included in our study were from our previous study (GSE133774), we have performed batch correction using Combat function from sva package (sva version). PCA plots (scale = TRUE) were generated to understand clustering with CpGs underlying whole genome and human ICRs. Hierarchical clustering was performed and visualized as heatmap using pheatmap package with “Euclidean” distance and “Ward.D” clustering method. Violin plots were created using ggplot2 (3.2.1). Heatmap was plotted using pheatmap (v.1.0.12). The raw and processed files are deposited in GEO under the accession GSE153211. Methylation level of imprinted DMRs was calculated as average of the methylation levels of their respective CpGs. Hypo- and hypermethylation of the DMRs were indicated if methylation level exceeded ± 2 standard deviation from average of 12 controls.

### DNA sequencing

#### Whole-exome sequencing analysis

Whole-exome sequencing was performed on genomic DNA of the probands and their parents (II-1, II-2, III-1, III-2) of family 1, I-4 of family 2, and I-2 of family 3. The DNA samples were sequenced 150 bp pair-end at BIODIVERSA srl Service (Milan, Italy) using the Agilent SureSelect V6 + UTR (~ 89 Mbp target) library and the Illumina NovaSeq6000 platform. The bioinformatic analysis was performed as previously reported [22]. In brief: reads were aligned to the human genome reference assembly (Genome Reference Consortium Human GRCh37) using the BWAmem software package v0.7.15 [33]. PCR duplicates were filtered out by Picard v2.9 (http://picard.sourceforge.net), and the GATK v3.7 suite was used to locally realign around inferred Insertion/Deletions (InDels) and recalibrate base quality scores. Single-nucleotide variants and InDels were called using GATK HaplotypeCaller and GenotypeGVCFs [34] and recalibrated with VariantRecalibrator. Recalibrated variants were annotated using wANNOVAR [35]. Genome variants with low coverage (< 15) or low quality (< 20) or in VQSRTrancheSNP99.00to99 or frequently occurring in general population (MAF > 0.01 in 1000 Genomes Project [36] or Exac (http://exac.broadinstitute.org/) [37] or gnomAD [38]) were filtered out.

The effect of the variants was predicted using the sequence-based tool PolyPhen-2 (http://genetics.bwh.harvard.edu/pph2) [39], whose scores correlate with the residual activity of the protein affected by the mutation [40], as well as Site Directed Mutator (SDM) that analyzes if the variants occurring at specific structural environment are tolerated within the family of homologous [41]. Template-based modeling was used to obtain a 3D structural model of human PADI6, due to the lack of crystal structure for this protein. The sequence was retrieved from the UniProt database (UniProt ID: Q6TGC4). Residues 10–694 of PADI6 could be aligned to protein-arginine deiminase type-4 (PADI4). A 3D model of a PADI6 dimer was built with SWISS-MODEL [42] in the automated mode. The program generated a list of the best possible templates. We chose 4dkt [43], which is the X-ray structure of PADI4, and built the model. Amino acids from 10 to the carboxy-terminus 694 could be modeled. The accessible surface area (ASA) was calculated with the protein interfaces, surfaces, and assemblies service PISA at the European Bioinformatics Institute [44].

#### Sanger sequencing

About 100 ng of genomic DNA was amplified by PCR and then sequenced by Sanger sequencing (Eurofins Genomics) to obtain the genotypes. PCR primers are listed in Additional file 11: Table S7.

### Web resources

The following web resources were used:

PolyPhen-2, http://genetics.bwh.harvard.edu/pph2

GnomAD, http://gnomad.broadinstitute.org/

SDM, http://marid.bioc.cam.ac.uk/sdm2

PISA, http://www.ebi.ac.uk/pdbe/prot_int/pistart.html

## Supplementary information

**Additional file 1: Table S1.** Pathogenic variants of *PADI6* and corresponding clinical phenotype.

**Additional file 2: Figure S1.** Pyrosequencing analysis of seven imprinted DMRs.

**Additional file 3 Figure S2.** Batch effect adjustment of array datasets.

**Additional file 4: Figure S3.** Violin plots showing whole-genome DNA methylation profiles of probands, their siblings and mothers, and 12 controls.

**Additional file 5: Table S2.** Methylation defects of imprinted DMRs in patients affected by BWS-MLID whose mothers are carriers of loss of function mutations in *PADI6.*

**Additional file 6: Table S3.** Maternal genetic variants in homozigosity or compound heterozigosity identified by WES.

**Additional file 7: Figure S4.** Sanger sequencing validating the *PADI6* variants identified by WES.

**Additional file 8: Table S4 .** List of the maternal-effect gene variants associated with MLID in the offspring identified so far and corresponding clinical phenotype.

**Additional file 9: Table S5.** Primers used for pyrosequencing analysis.

**Additional file 10: Table S6.** Sex and age information of controls of methylome analysis.

**Additional file 11: Table S7.** Primers for Sanger sequencing validation.

## Data Availability

Methylation array data that support the findings of this study have been deposited under accession code GSE153211 in the Gene Expression Omnibus repository.

## Abbreviations

BWS: Beckwith-Wiedemann syndrome
DMRs: Differentially methylated regions
gDMR: Germline-derived differentially methylated region
GOM: Gain of methylation
ICs: Imprinting centers
IDs: Imprinting disorders
LOM: Loss of methylation
MLID: Multi-locus imprinting disturbance
PBL: Peripheral blood leukocyte
PCA: Principal component analysis
SCMC: Subcortical maternal complex
SRS: Silver-Russell syndrome
SNV: Single-nucleotide variant
TNDM: Transient neonatal diabetes mellitus
UPD: Mosaic segmental paternal unidisomy
WES: Whole-exome sequencing
