# Supplementary figures and images for: Loss-of-function maternal-effect mutations of PADI6 are associated with familial and sporadic Beckwith-Wiedemann syndrome with multi-locus imprinting disturbance
Source: Clin Epigenetics. 2020 Sep 14;12:139. doi: 10.1186/s13148-020-00925-2 (PMC7489023; doi:10.1186/s13148-020-00925-2)

**a**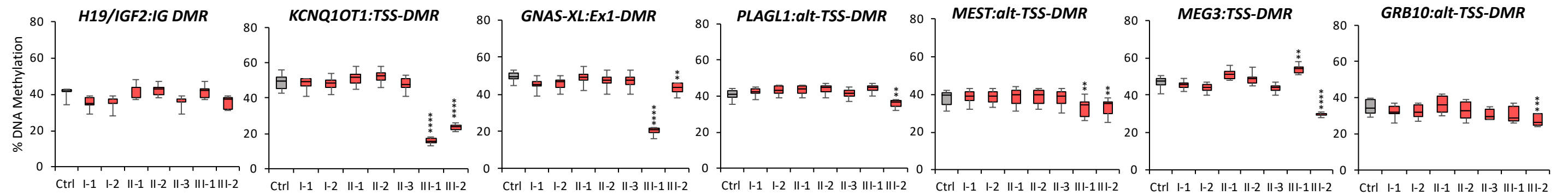**b**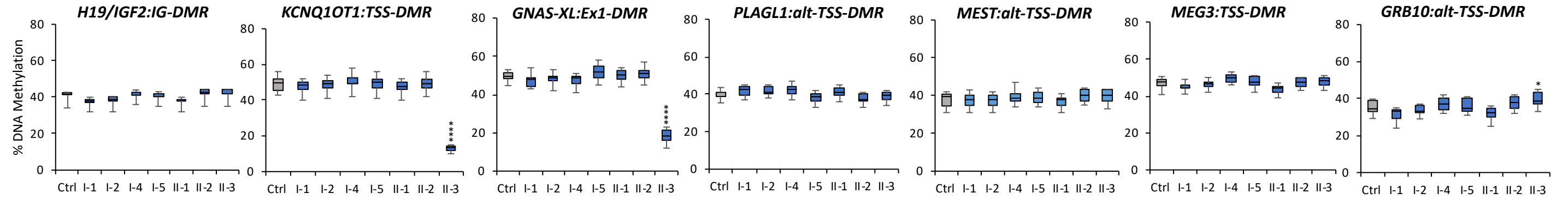**c**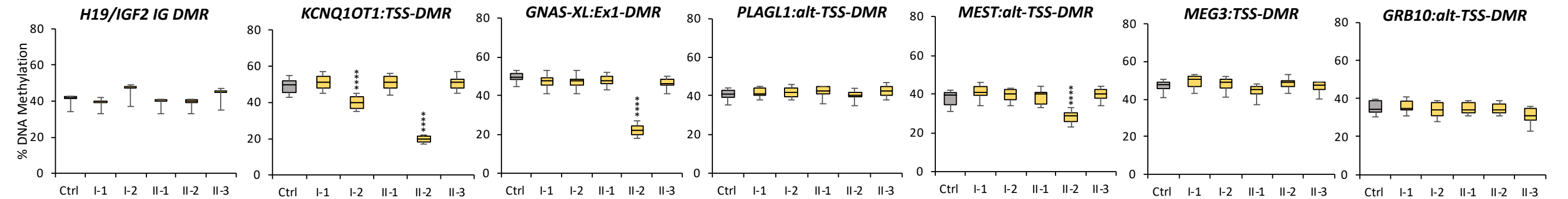

Supplement: Supplementary file 2 — Additional file 2: Figure S1. Pyrosequencing analysis of seven imprinted DMRs. [file 13148_2020_925_MOESM2_ESM.pdf]

**a**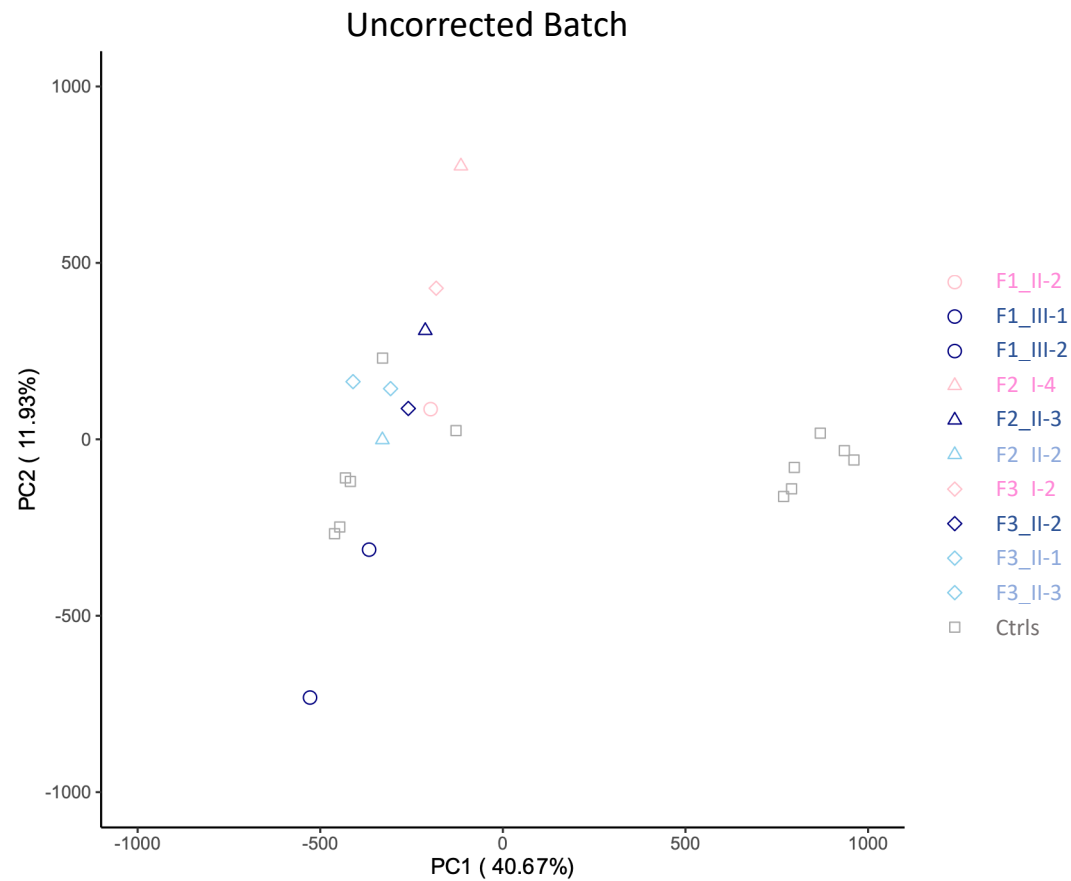**b**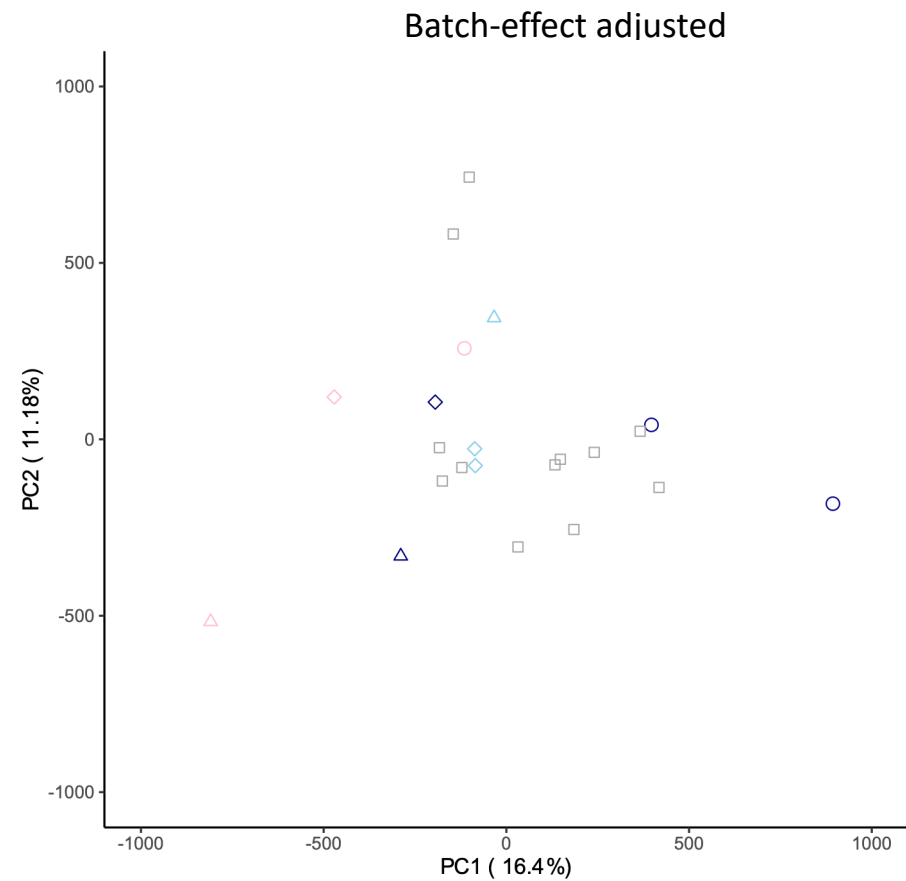

Supplement: Supplementary file 3 — Additional file 3 Figure S2. Batch effect adjustment of array datasets. [file 13148_2020_925_MOESM3_ESM.pdf]

## whole-genome

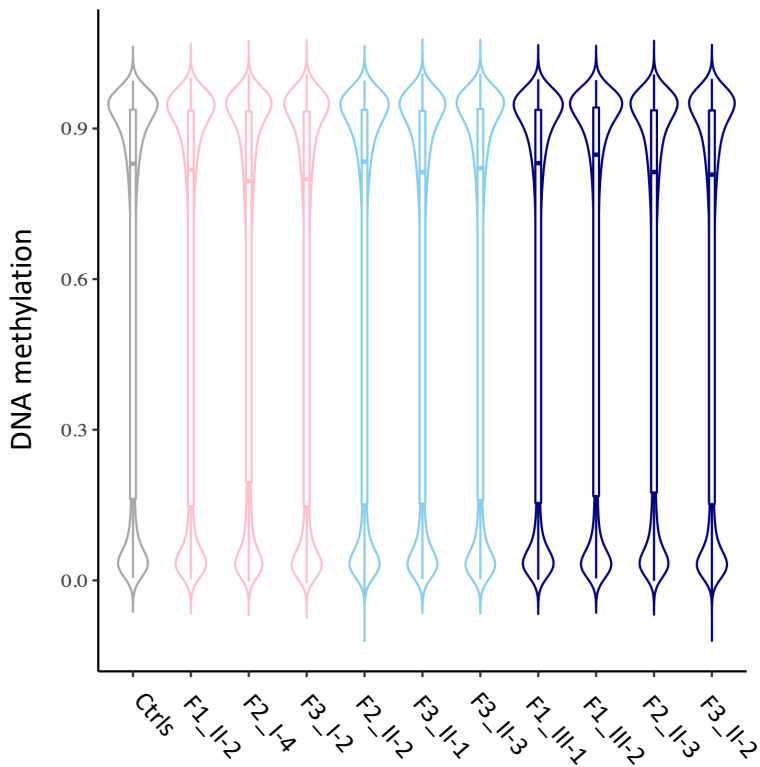

Supplement: Supplementary file 4 — Additional file 4: Figure S3. Violin plots showing whole-genome DNA methylation profiles of probands, their siblings and mothers, and 12 controls. [file 13148_2020_925_MOESM4_ESM.pdf]

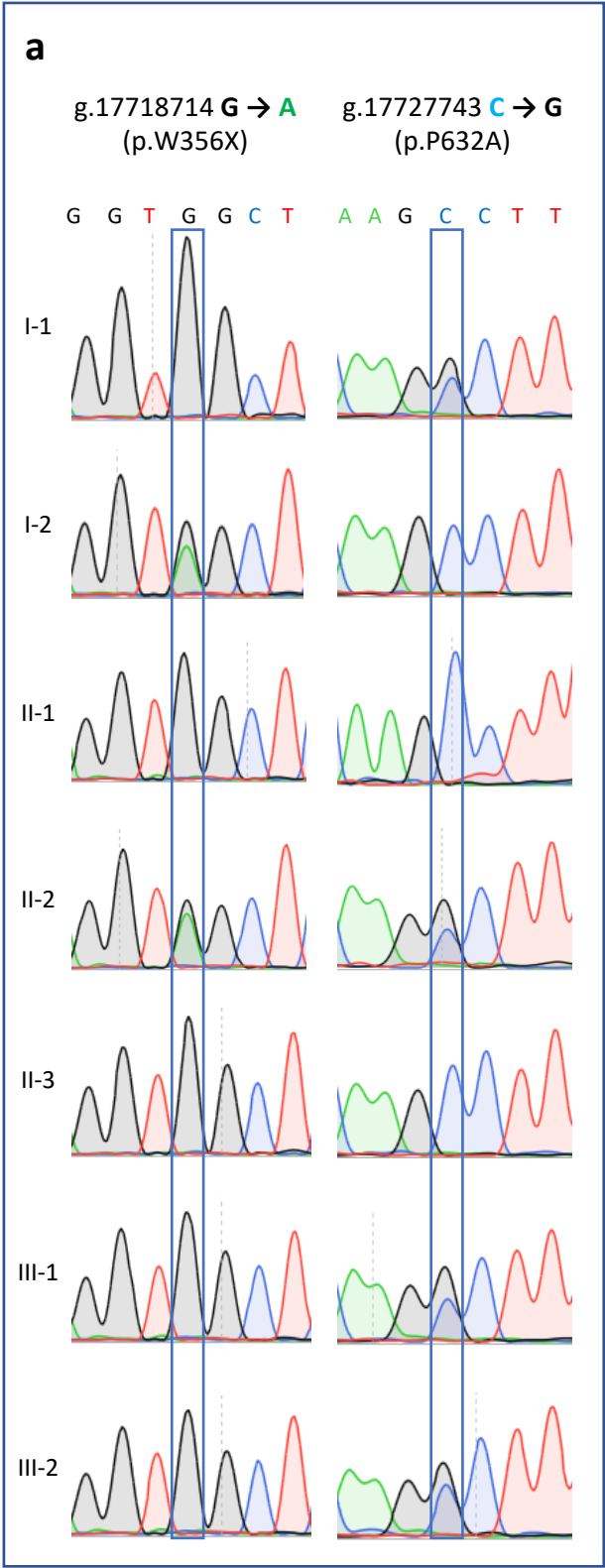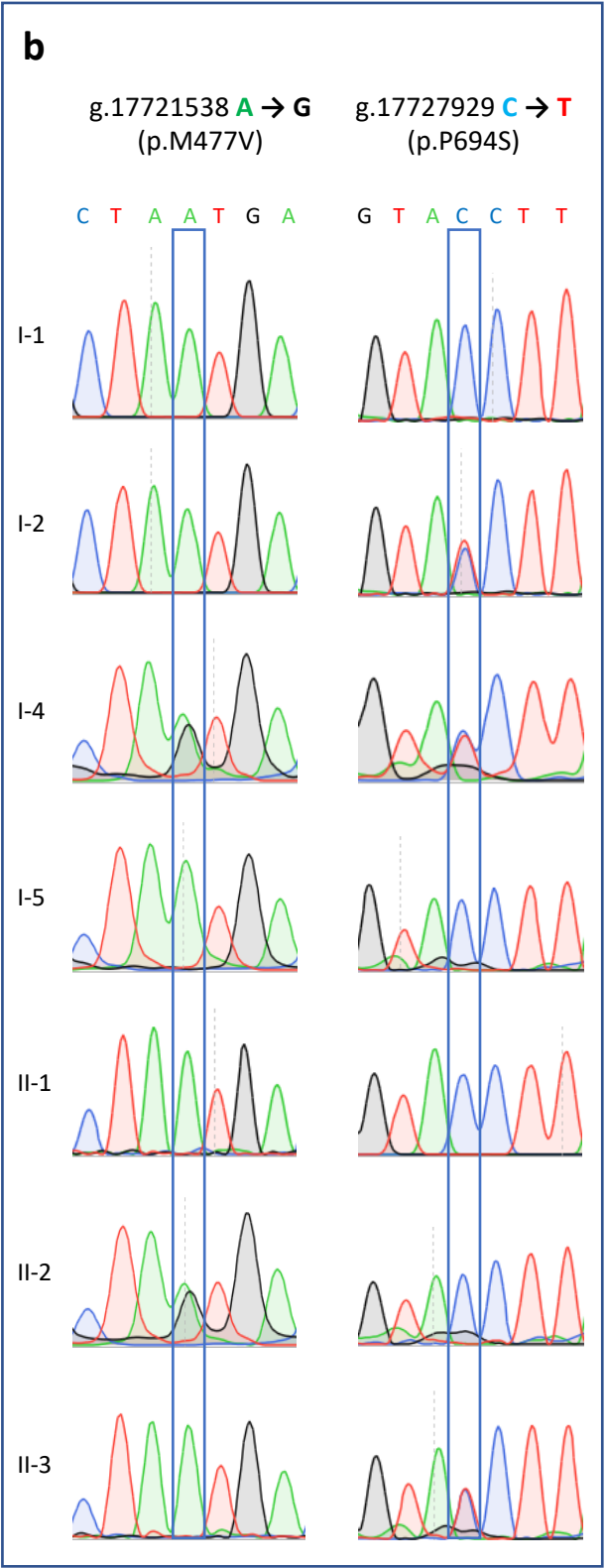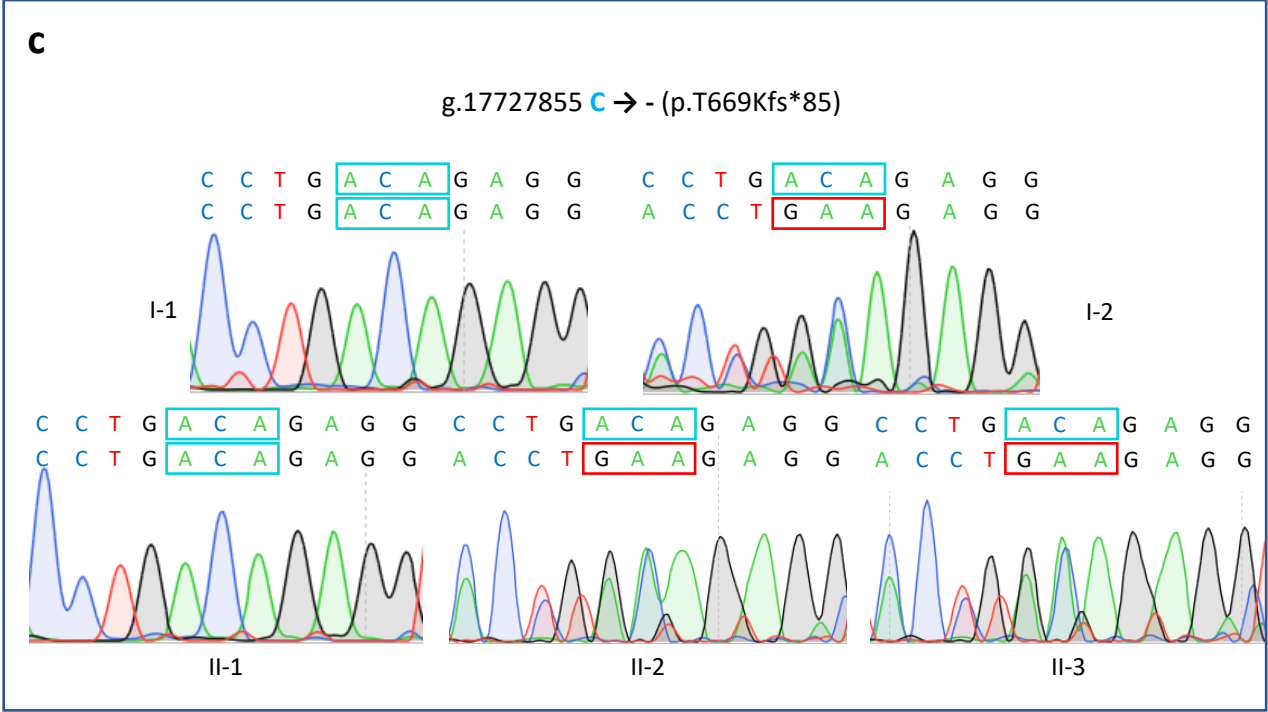

Supplement: Supplementary file 7 — Additional file 7: Figure S4. Sanger sequencing validating the PADI6 variants identified by WES. [file 13148_2020_925_MOESM7_ESM.pdf]
