# Supplementary material for: Loss-of-function maternal-effect mutations of PADI6 are associated with familial and sporadic Beckwith-Wiedemann syndrome with multi-locus imprinting disturbance
Source: Clin Epigenetics. 2020 Sep 14;12:139. doi: 10.1186/s13148-020-00925-2 (PMC7489023; doi:10.1186/s13148-020-00925-2)
Supplement: Supplementary file 9 — Additional file 9: Table S5. Primers used for pyrosequencing analysis. [file 13148_2020_925_MOESM9_ESM.docx]

| **ICR** | **Primers** | **CpGs** | **Genomic Coordinates**  **(hg19)** |
| --- | --- | --- | --- |
| ***H19/IGF2*:IG-DMR** | Forward:  5′-Biotin/ GTGGTTTTTATGACTGTTTTATTTTTGATGA-3′ | 5 | Chr11:  2,021,880-2,022,088 |
|  | Reverse:  5′-ACTTCCCCTTCAATCTCACCA -3′ |  |  |
|  | Sequencing:  5′-TACAAAATTAATTATAACTATAAAAT-3′ |  |  |
| ***KCNQ1OT1*:TSS-DMR** | Forward:  5′-GGAGAGTATTGTTTAGGTTAGGTTGTAT -3′ | 9 | Chr11:  2,720,485-2,720,644 |
|  | Reverse:  5′ Biotin/CCTCCCCATCTCTCTAAAAAAATTTAA-3′ |  |  |
|  | Sequencing:  5′-GGTTAGGTTGTATTGTTG-3′ |  |  |
| ***PLAGL1*:alt-TSS-DMR** | Forward:  5'-GTTAAGTGGTAGGAGGAGGTTT-3' | 11 | Chr6:  144,329,595-144,329,725 |
|  | Reverse:  5′-Biotin/CTATACCTAAACCACCTTAACTTTACCC-3' |  |  |
|  | Sequencing:  5′-GGTAGGAGGAGGTTT-3′ |  |  |
| ***GNAS-XL*:TSS-DMR** | Forward:  5'-GGTAGTTTATTTTAAGAGGTTGTTAGAT -3' | 6 | Chr20:  57,429,164-57,429,411 |
|  | Reverse:  5′-Biotin/ACTCCTTCCATCTCTACTACTT-3' |  |  |
|  | Sequencing:  5-GATATTTTAGTTAATATGGATAGTT-3' |  |  |
| ***MEST:alt-*TSS-DMR** | Forward:  5'-Biotin/AATAAAGGGGGTTTTGTTTTTTTAAT-3' | 13 | Chr7:  130,131,089-130,131,334 |
|  | Reverse:  5'-AACCCACCACCAAACTAAT-3' |  |  |
|  | Sequencing:  5'-TAACCACTATAACCAAAATTAC-3' |  |  |
| ***MEG3:TSS*-DMR** | Forward:  5'- Biotin/GTTTATTTAAGAGGGAATAGTTTTGAGAT-3' | 8 | Chr14:  101,293,753-101,293,946 |
|  | Reverse:  5'- CCTCTCTCTCCATCCTACTCA-3' |  |  |
|  | Sequencing:  5'- AAAACCACTAAAAATCAACT -3' |  |  |
| ***GRB10*:alt-TSS-DMR** | Forward:  5'- Biotin/GGTAGGGGTTTTTGTAGTTTG-3' | 9 | Chr7:  50,850,014-50,850,128 |
|  | Reverse:  5'-CTCTCCAAATACTCAAATAAACTC-3' |  |  |
|  | Sequencing:  5'- CCAAATACTCAAATAAACTCC-3' |  |  |

**Table S5.** Primers for pyrosequencing analysis.
