# Supplementary material for: Loss-of-function maternal-effect mutations of PADI6 are associated with familial and sporadic Beckwith-Wiedemann syndrome with multi-locus imprinting disturbance
Source: Clin Epigenetics. 2020 Sep 14;12:139. doi: 10.1186/s13148-020-00925-2 (PMC7489023; doi:10.1186/s13148-020-00925-2)
Supplement: Supplementary file 10 — Additional file 10: Table S6. Sex and age information of controls of methylome analysis. [file 13148_2020_925_MOESM10_ESM.docx]

| Controls | Gender | Age |
| --- | --- | --- |
| Ctrl_1 | Male | 17 |
| Ctrl_2 | Male | 16 |
| Ctrl_3 | Female | 32 |
| Ctrl_4 | Female | 25 |
| Ctrl_5 | Male | 15 |
| Ctrl_6 | Female | 15 |
| Ctrl_7 | Female | 44 |
| Ctrl_8 | Female | 26 |
| Ctrl_9 | Female | 2 |
| Ctrl_10 | Male | <10 |
| Ctrl_11 | Female | <10 |
| Ctrl_12 | Male | <10 |

**Table S6.** Sex and age information of controls of methylome analysis.
