# Supplementary material for: Loss-of-function maternal-effect mutations of PADI6 are associated with familial and sporadic Beckwith-Wiedemann syndrome with multi-locus imprinting disturbance
Source: Clin Epigenetics. 2020 Sep 14;12:139. doi: 10.1186/s13148-020-00925-2 (PMC7489023; doi:10.1186/s13148-020-00925-2)
Supplement: Supplementary file 11 — Additional file 11: Table S7. Primers for Sanger sequencing validation. [file 13148_2020_925_MOESM11_ESM.docx]

| **Variant** | **Primers** | **Coordinates of the**  **amplified region in hg19** |
| --- | --- | --- |
| **W356X** | Forward:  5′- CTGCAGGGTTTTGTGGACAC -3′ | chr1:17,718,619-17,718,776 |
|  | Reverse:  5′- CATGCTGAGTCTCCCCACC -3′ |  |
| **M477V** | Forward:  5′- AGACTTCCTCTATGCCCAGC -3′ | chr1:17,721,480-17,721,660 |
|  | Reverse:  5′- AGATTCCCCAACAGAAGGCA -3′ |  |
| **P632A** | Forward:  5′- CTCACCCACCCTGTCTTTCT -3′ | chr1:17,727,676-17,727,827 |
|  | Reverse:  5′- AGGTGCACTTGAAGCCCA -3′ |  |
| **T669Kfs*85** | Forward:  5′- GGCTTCAAGTGCACCTTCAT -3′ | chr1:17,727,812-17,727,988 |
|  | Reverse:  5′- GGTGACAGTGGGCCATCC -3′ |  |
| **P694S** | Forward:  5′- GGCTTCAAGTGCACCTTCAT -3′ | chr1:17,727,812-17,728,052 |
|  | Reverse:  5′- GAAAGGGTTCTGTTGCCTGG -3′ |  |

**Table S7.** Primers for Sanger sequencing validation.
